# Integrated sources of photon quantum states based on nonlinear optics

**DOI:** 10.1038/lsa.2017.100

**Published:** 2017-11-17

**Authors:** Lucia Caspani, Chunle Xiong, Benjamin J Eggleton, Daniele Bajoni, Marco Liscidini, Matteo Galli, Roberto Morandotti, David J Moss

**Affiliations:** 1 Institute of Photonics, Department of Physics, University of Strathclyde, Glasgow G1 1RD, UK; 2 Institute of Photonics and Quantum Sciences, Heriot-Watt University, Edinburgh EH14 4AS, UK; 3 Centre for Ultrahigh bandwidth Devices for Optical Systems (CUDOS), Institute of Photonics and Optical Science (IPOS), School of Physics, University of Sydney, Sydney, NSW 2006, Australia; 4 Dipartimento di Ingegneria Industriale e dell’Informazione, Università di Pavia, via Ferrata 1, 27100, Pavia, Italy; 5 Dipartimento di Fisica, Università di Pavia, via Bassi 6, 27100 Pavia, Italy; 6 INRS-EMT, 1650 Boulevard Lionel-Boulet, Varennes, Québec J3X 1S2, Canada; 7 Institute of Fundamental and Frontier Sciences, University of Electronic Science and Technology of China, Chengdu 610054, China; 8 National Research University of Information Technologies, Mechanics and Optics, St. Petersburg, Russia; 9 Center for Microphotonics, Swinburne University of Technology, Hawthorn, Victoria, 3122 Australia

**Keywords:** entanglement, integrated optics, nonlinear optics, photon pairs, quantum optics, quantum states

## Abstract

The ability to generate complex optical photon states involving entanglement between multiple optical modes is not only critical to advancing our understanding of quantum mechanics but will play a key role in generating many applications in quantum technologies. These include quantum communications, computation, imaging, microscopy and many other novel technologies that are constantly being proposed. However, approaches to generating parallel multiple, customisable bi- and multi-entangled quantum bits (qubits) on a chip are still in the early stages of development. Here, we review recent advances in the realisation of integrated sources of photonic quantum states, focusing on approaches based on nonlinear optics that are compatible with contemporary optical fibre telecommunications and quantum memory platforms as well as with chip-scale semiconductor technology. These new and exciting platforms hold the promise of compact, low-cost, scalable and practical implementations of sources for the generation and manipulation of complex quantum optical states on a chip, which will play a major role in bringing quantum technologies out of the laboratory and into the real world.

## Introduction

Quantum mechanics underpins many of the scientific and technological advancements that have already had a significant impact on our society, ranging from ultrafast computing to high-sensitivity metrology and secure communications. Furthermore, it holds the promise of profound future innovations that will redefine many areas, such as quantum computing, offering unprecedented computational power, as well as emerging technologies such as non-classical imaging and spectroscopy, where quantum mechanics offers a means to greatly increase sensitivity. In particular, the field of quantum telecommunications is already providing ultimate communications security that is directly guaranteed by the laws of physics rather than by complex mathematical algorithms.

Most of these technologies exploit the peculiar properties of quantum mechanics, such as the principles of superposition and entanglement. Superposition allows a quantum system to be in two different states simultaneously, while a quantum system composed of more than one component (for example, particles or photons) is said to be entangled if it can only be described as a whole (see Supplementary Section A).

While many different physical systems have been exploited for quantum technologies, including trapped ions and semiconductor circuits, photonics has played a particularly crucial role^1, 2, 3^. Historically, light and its ultimate constituent – the photon, or the quantum of light – have served as a testing ground for many breakthrough experiments aimed at confirming the apparent counterintuitive nature of quantum mechanics. This was highlighted by the seminal work on the violation^4^ (and more recently, loophole-free violation^5, 6^) of Bell’s inequalities, which demonstrated the non-local character of quantum mechanics, a fundamental property that cannot be explained by hidden-variables theories, as suggested 40 years earlier by Einstein, Podolsky and Rosen^7^.

Photonics has become a widespread platform in quantum experiments for several reasons: i) the possibility of easily transmitting quantum states encoded in a photon by means of free space optical links or through fibre optic networks; ii) the advances in nonlinear optics that have enabled the generation of single and entangled photons; and iii) the lack of extreme sensitivity to environmental noise (thermal, electromagnetic, etc.) that plagues solid-state approaches. Nonlinear parametric processes have been instrumental in generating fundamental quantum states of light. When an intense pump laser field propagates through a nonlinear medium, there is a probability that two new photons are generated as a pair, either as uncorrelated photons or in an entangled state.

The ability to achieve these functions on photonic integrated chips or circuits is absolutely key to moving quantum technologies out of the laboratory and into the real world. The main components of quantum photonic systems, such as mirrors, beam splitters, and phase shifters, are all now realisable in an integrated form^8, 9^. Ultimately, all functions needed for quantum demonstrations – the generation, manipulation and detection of single/entangled photons – would ideally be integrated in just one chip^10^. However, even just the ability to integrate one function, such as the source of non-classical light, would already offer many advantages over bulk optical setups.

Here, we review recent advances in integrated, or chip-based, sources of quantum states of light, including single and entangled photons, and the techniques for characterising heralded and entangled photon sources. We focus on devices based on nonlinear optics that are compatible with electronic on-chip technology complementary metal oxide semiconductor (CMOS), ending with a discussion on recent achievements in the generation of single photons on demand. We refer the reader elsewhere for other relevant results based on integrated chips, for example, quantum states^11, 12, 13, 14^, quantum interference^15, 16, 17, 18, 19, 20, 21^, quantum logic ports^12, 22, 23^, quantum algorithms^24^, quantum walks^25, 26, 27, 28, 29^, and boson sampling^30, 31, 32, 33, 34^, as well as reviews on related topics, including quantum metrology^35^, computing^36^, integrated detectors, typically superconducting nanowires^37, 38^ in different platforms (for example, GaAs^39^, silicon-on-insulator^40^, diamond^41^ and silicon nitride^42^) and a more general range of sources^9, 14, 43, 44, 45, 46, 47, 48, 49, 50, 51^.

## Entangled and single-photon sources

The key states of interest for quantum photonic devices are single and entangled photons. These can be both produced via spontaneous nonlinear parametric processes. Depending on the platform material, these occur via second- (χ^(2)^) or third-order (χ^(3)^) nonlinearities, where either one (for χ^(2)^) or two (for χ^(3)^) photons from an intense pump laser are annihilated into two daughter photons. The χ^(2)^ process is termed spontaneous parametric down-conversion (SPDC), while the χ^(3)^ process is called spontaneous four-wave mixing (SFWM). These processes are the quantum counterparts of classical difference-frequency generation and four-wave mixing (FWM), respectively. In the non-classical case, the seed fields are provided by vacuum fluctuations: only the virtual signal and idler pairs that satisfy energy and momentum conservation are efficiently transformed into real photons. Alternatively, we can think of SPDC as a photon fission process, while SFWM is more of an elastic scattering process.

One of the main differences between SPDC and SFWM is that for SPDC, energy conservation requires the signal and idler daughter photons to be generated at frequencies that are symmetrically located with respect to half of the pump field frequency, while in SFWM, they are symmetrically distributed around the pump frequency:


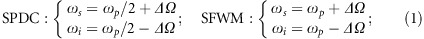


where ω_*p*_, *ω*_*s*_, and *ω*_*i*_ represent the pump, signal, and idler frequencies, respectively, while *ΔΩ* is the frequency shift with respect to the degenerate process. This implies that in SFWM, all of the involved fields can have similar wavelengths. While this can be useful in satisfying phase matching conditions (momentum conservation), it also increases the difficulty in filtering out the pump to isolate the signal and idler photons.

### Entangled photons

The combination of vacuum fluctuations and conservation laws is at the core of the entanglement between signal and idler photons. Depending on the configuration of the conversion process, entanglement can be generated in different degrees of freedom, for example, polarisation, space, time, and orbital angular momentum, and is a fundamental resource for quantum computing and communications. Indeed, many quantum algorithms rely on entanglement^52^.

To achieve entanglement, the signal and idler photons need to be generated in at least a two-mode state, for example, with horizontal and vertical polarisations. For type I SPDC, the signal and idler photons are always generated with the same polarisation, for example:


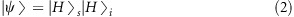


whereas for type II SPDC, they are generated with orthogonal polarisations, and it is thus possible to obtain, for example, the entangled state:


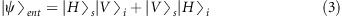


More formally, the two cases are referred to as one- and two-mode squeezing transformations.

Protocols based on entanglement have been proposed (for example, the E91 protocol^53^) for applications in quantum cryptography, where ‘Alice’ and ‘Bob’ each share a component of a bipartite entangled state. Eavesdropping can be detected by exploiting the collapse of the wave function upon measurement. The multimode nature of the relevant variable provides the alphabet for the exchange of a cryptographic key. The higher the dimensionality of the state, the larger the amount of information each qubit can contain. Different degrees of freedom have been investigated for this purpose, for example, space^54^, time^3, 55^ (or its conjugate variable, frequency^56^) and orbital angular momentum^57^.

### Heralded single photons

A single photon is a particular quantum state where one and only one photon is present, and it is fundamental for quantum information and computing. One of the most widespread quantum cryptographic protocols, the BB84^58^, relies on single photons, where security is provided by the fact that i) it is not possible to measure the quantum state of a system without perturbing it; ii) a single photon cannot be partially measured since it is the ultimate quantum of electromagnetic radiation; and iii) it is not possible to perfectly clone an unknown quantum state (no-cloning theorem^59, 60^). In 2000, a universal quantum computing approach based on single photons and linear optics^61^ was proposed, commonly referred to as linear optical quantum computing (LOQC). For all these applications, there is a great need for more efficient and reliable single-photon sources.

Such sources can be distinguished according to whether they are deterministic or probabilistic, depending on whether the photons are available ‘on demand’ or at an unknown time, respectively. For cryptography or computing, deterministic sources are much more preferable and these are discussed in Section Deterministic sources below.

In both SPDC and SFWM, the signal and idler photons are always emitted in pairs and correlated in time. This correlated emission, while probabilistic, can be exploited in a heralding scheme where one photon signals the presence of the other, although this approach is limited by both loss and multiple pair generation. Each time a signal or idler photon is lost, either no heralding occurs, and thus the single photon is present but not usable, or vice versa – an empty state is heralded. The state generated by spontaneous parametric processes can in general be expressed as^62^:


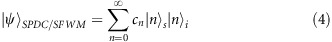


where *n* is an integer number, *s* and *i* represent signal and idler, respectively, and 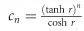 represents complex coefficients, with *r* being a squeeze parameter that depends on the pump intensity (and determines the average photon number 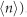. The probability to find exactly *n* photons in the signal and *n* photons in the idler is given by 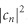. For vacuum squeezed states, the photon number distribution 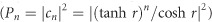 is maximum at *n*=0, while for other states, such as coherent states, the photon number distribution peaks at 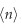. If the parameter *r* is small enough (that is, if the pump intensity is sufficiently low), only the first two terms are relevant, corresponding to either no generation or the generation of a single pair. If multiple pairs are created, at least two photons are simultaneously present in each beam, which can result in the heralding of more than one photon, in turn compromising, for example, quantum cryptography security. As a rule of thumb, the pump intensity should be kept low enough to have an average of no more than 0.1 signal/idler pairs per pump pulse (or per pump coherence time in the case of continuous wave excitation). While this low-gain regime is necessary for heralded single-photon sources, quantum entanglement between signal and idler fields can also be preserved in the high-gain regime, where very intense beams can be generated, as in the case of intensity/phase entanglement in twin beams^63^. By judicious engineering of a probabilistic source, for example, by properly combining different SPDC or SFWM processes, an almost deterministic single-photon source can be realised (see Section Deterministic sources below).

## Characterising a heralded single-photon source

### True single photons

The key issue with heralded single-photon sources is whether or not the heralded state is indeed a single photon. This is typically determined by measuring the degree of second-order coherence, or the 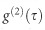 function^62, 64^, that characterises the photon statistics of a field and that is related to its temporal intensity fluctuations via:


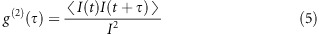


where *I(t)* is the field intensity at time *t* (defined as the average over many field oscillations). It can be measured, for example, by splitting a beam using a 50/50 splitter and then recording the intensity correlations at the output ports as a function of the relative delay (Hanbury-Brown and Twiss, or intensity interferometer).

**Figure 1:**
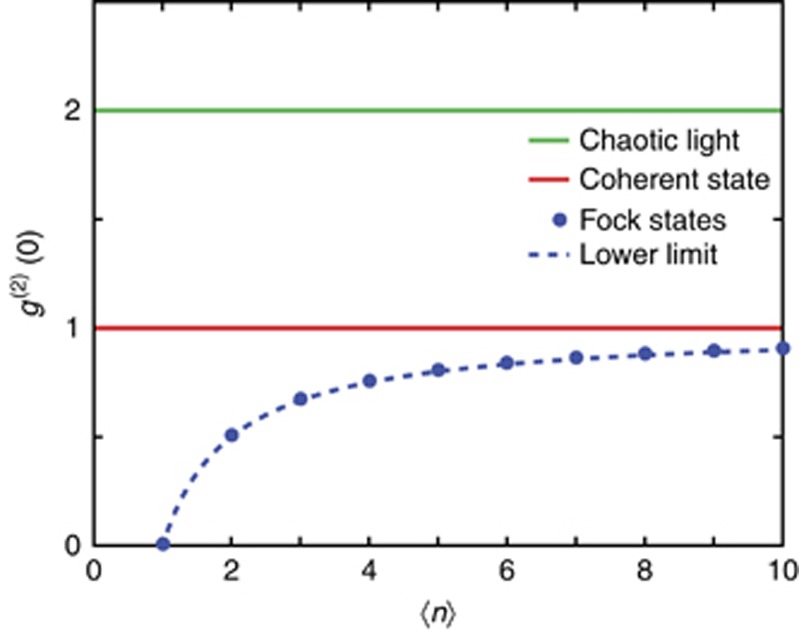
Value of 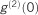 for different states as a function of the average photon number 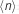: chaotic or thermal light (green line), coherent state (red line), and Fock states (blue dots). The dashed blue line represents the lower limit for 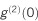 in the quantum treatment.

Classically, the value at zero delay is 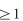, that is, 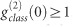. However, in the quantum treatment, the operator character of the fields must be taken into account; this allows one to access an additional range of values below unity. For example, for Fock, or number, states composed of an exact number of photons (without any intensity fluctuations), we have:


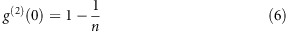


where *n* is the number of photons. A plot of 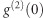 for different states is shown in Figure 1.

**Figure 2:**
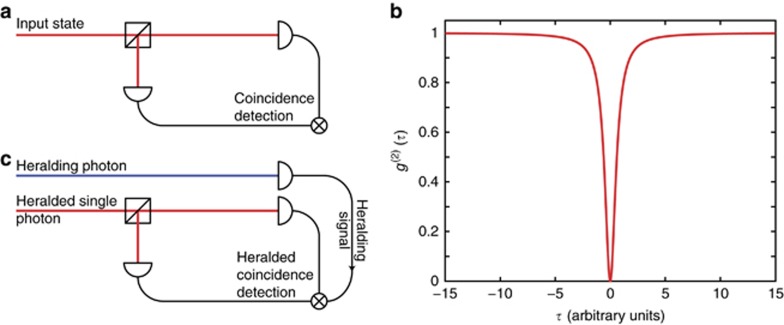
(**a**) Characterisation of a single-photon state. The beam is divided by a beam splitter, and the coincidences between the output ports are recorded as a function of the relative delay *τ*. (**b**) Expected second-order coherence function for a single-photon state. At zero delay, we have a dip reaching zero. Note that the shape and width of the function are arbitrary and in general depend on the particular process considered for generating the single photons. (**c**) Characterisation of a heralded single-photon source. In this case, the coincidences between the output ports of the beam splitter are measured if and only if the detector on the heralding arm fires.

For a perfect single-photon source, 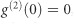, which can be intuitively understood by considering a single photon entering a 50/50 beam splitter (Figure 2a). Since a single photon is the ultimate quantum of radiation, it cannot be split further; thus, it can only exit one port of the beam splitter, not both. Therefore, the number of coincidences at the output ports of a beam splitter, as a function of the relative arrival time of photons, displays a dip at zero delay (Figure 2b). At large delays, 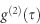 approaches unity, regardless of the photon state. The closer the dip is to zero at zero delay, the better the source approaches a true single-photon source. In general, for realistic sources, 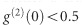
is required to claim a single-photon state since the theoretical value of 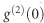 for a two-photon Fock state is 0.5.

For a heralded single-photon source, the characterisation setup is very similar, but the coincidences at the beam splitter output are only measured when the heralded photon is detected (Figure 2c).

### Purity of the state

In general, a fundamental requirement for a single-photon source is the purity of the generated state. Indeed, many quantum information applications (for example, LOQC gates^65^) are based on the interference of two or more single photons and require pure states for optimal visibility. Thus, unentangled photons are generally required since this is a necessary condition to herald single photons in a pure state^66^. This situation is in contrast to the generation of entangled photons (see Section Entangled photons above), in which quantum correlations are not only desired, but in fact are a fundamental requirement.

The purity of a single-photon state can be measured using different techniques. The most formal techniques rely on measuring the density matrix of the state, 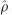, using the purity obtained from the trace of the density matrix squared: 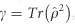, where *γ*=1 refers to a pure state. Generally, this is the most complete characterisation of a quantum state, as it contains all the relevant information for both single photons and entangled states^67, 68^. However, determining 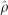 requires several different measurements. For example, for a *D*-dimensional, *n*-partite (for example, composed of *n* photons) quantum system, 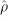 is represented by a *D*^*n*^ × *D*^*n*^ complex matrix. Considering that the density matrix is normalised and Hermitian, that is, the conditions 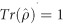 and 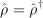 must hold, it is implied that, in general, *D*^*2n*^-1 parameters must be identified. These parameters can be obtained by taking a set of *D*^*2n*^ different projection measurements^69^. For example, the state of 2 polarisation-entangled qubits can be characterised by measuring the coincidences in 16 different combinations of the two photon polarisation states (for example, all combinations of the horizontal, vertical, +45°, and right circular polarisation settings)^69^. Similarly, the full characterization of 3-photon polarisation-entangled states require one to measure triple coincidence events in 64 different settings, and so on.

An alternative approach relies on demonstrating that the source is single mode, since in this case the measurement of the heralding photon will project the single photon into the corresponding pure single mode^70^ (see Supplementary Section B). Note also that the normalisation condition 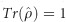 combined with the purity condition 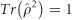 implies that for a pure state, the diagonalization of the density matrix leads to only 1 non-zero eigenvalue, that is, a pure state can always be represented by a single-mode state in the proper basis. A single-mode photon can be obtained via a multimode generation process, provided that suitable filtering is applied before detection, although at the expense of reducing the efficiency of the source. Alternatively, single-mode emission can be obtained by modifying the process parameters, such as the pump spectrum and phase matching curve (see Chapter 11.2.4. in Ref. 71 for details on heralding pure single-photon states).

**Figure 3:**
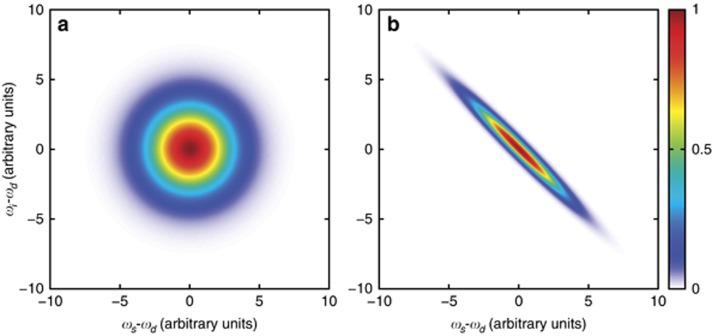
Examples of normalised joint spectral density for frequency-uncorrelated (**a**) or frequency-correlated (**b**) signal and idler photons. The axes represent the frequency shift with respect to degeneracy (*ω*_*d*_ =*ω*_*p*_/2 and *ω*_*d*_=*ω*_*p*_ for SPDC and SFWM, respectively) for signal (*x*-axis) and idler (*y*-axis) photons.

The number of modes can be obtained directly by measuring the signal-idler correlations for a specific variable. For example, the single- or multimode character in the frequency domain can be determined by measuring the signal/idler joint spectral distribution (JSD), that is, the frequency of the idler given the frequency of the signal. Single-mode emission will then be characterised by uncorrelated signal and idler photons (Figure 3a), while correlation is an indication of a multimode character (Figure 3b). The JSD can be obtained by measuring, for each idler frequency, the coincidences for all the signal frequencies. This measurement is typically obtained by exploiting narrowband filters (able to resolve the frequency bandwidth over which the signal and idler photons are generated), although this typically introduces significant loss, particularly for very narrow bandwidths. In turn, this can jeopardise the whole measurement by requiring extremely long integration times to compensate for losses. A possible solution is to exploit the corresponding SPDC and SFWM stimulated processes^72, 73^, for example, by providing as the input the signal field at different frequencies and measuring the idler power. The stimulated process avoids the need for single-photon detectors and strongly reduces the measurement time. This is particularly suitable for characterising states generated by integrated resonators, where the very narrow linewidth requires resolutions of picometres or less and low loss filters are generally not available. Finally, by exploiting the known statistics of the separate signal and idler beams, one can avoid the need for filtering the signal and idler fields, useful for very narrow linewidths. In SPDC and SFWM, signal and idler beams individually exhibit thermal statistics as a result of the amplification of vacuum fluctuations. In turn, the number of modes of a thermal state can be measured based on the degree of second-order coherence, the zero-delay value of which is related to the number of modes through the relation^64, 74, 75^:


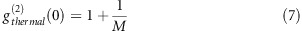


where *M* represents the total number of modes of all involved variables. Provided that all the modes are effectively coupled to the detector, this technique can resolve very narrow frequency modes. Indeed, this requires the temporal resolution of the detector (typically limited by jitter and being of the order of hundreds of picoseconds for telecom detectors) to be shorter than the photon coherence time (which, in turn, is quite long for narrow frequency bandwidth photons, for example, nanoseconds for hundreds of MHz bandwidth photons).

### Heralding probability

Another fundamental parameter is the heralding probability – the probability of measuring an idler photon once the heralding signal counterpart has been detected. This quantity is strictly related to the loss of the system from generation to detection, and for a lossless system, the probability is 100%. It is defined as^76^:


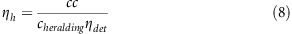


where *cc* denotes the coincidence counts, *c*_*heralding*_ denotes the single counts on the heralding arm (for example, signal), and *η*_*det*_ is the quantum efficiency of the detector on the heralded single-photon arm (idler). The heralding probability allows for a comparison of different sources independently of the specific detectors used.

### Coincidence to accidental ratio (CAR)

**Figure 4:**
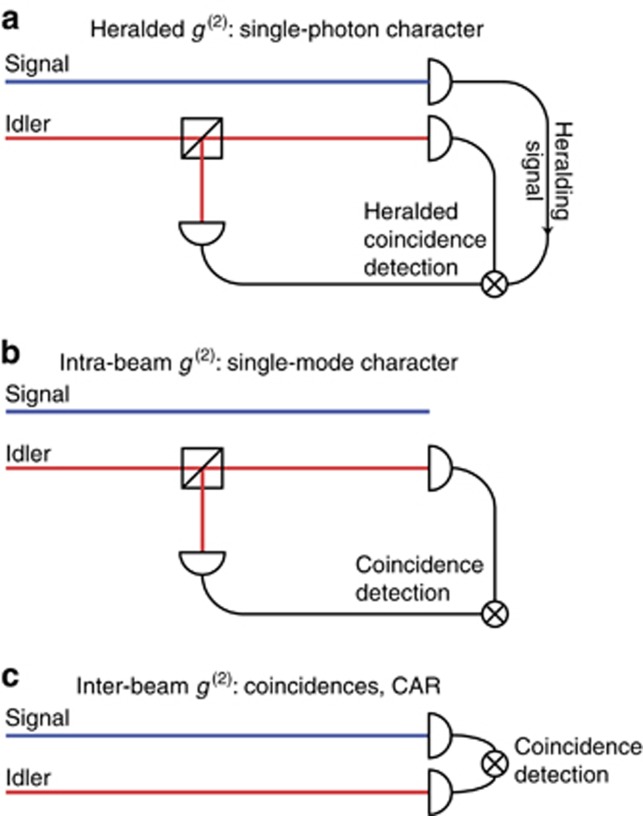
Comparison of the experimental setups for measuring the different types of *g*^(2)^ functions reported in this article: heralded *g*^(2)^ for investigating the single-photon character (**a**), intra-beam *g*^(2)^ for single-mode characterisation (**b**), and inter-beam *g*^(2)^ for coincidence and CAR measurements (**c**).

This parameter characterises how well the source generates photon pairs for both entangled pair and heralded photon sources. It is evaluated by measuring the coincidences between the signal and idler photons as a function of the relative delay (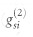, often referred to as inter-beam *g*^*(2)*^ or intensity cross-correlation (see Figure 4c)). In the ideal case, where signal and idler photons are emitted only in single pairs and without noise or loss, coincidences occur only near zero delay, with no coincidences at all for delays longer than the signal idler coherence time (*τ*_coh_ typically determined by the phase matching conditions for single-pass SPDC and SFWM, and by the cavity lifetime for cavity-enhanced processes). The CAR is often defined as:


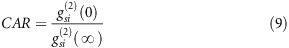


however, this overestimates the true CAR, and a more formal definition should take into account the finite size of the correlation peak^77^:


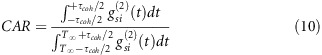


which represents the ratio between the sum of all coincidences within the peak and the sum of the coincidences over a temporal window of the same size far from the peak (*T*_*∞*_ is an arbitrary temporal delay far from the peak). In general, the CAR can be affected by loss, by multiple-pair generation, and by noise in the detectors^71, 78^. If competing emission processes, such as photoluminescence or Raman scattering, are absent, then the CAR is directly related to the probability of emitting multiple pairs^79^ and thus to the suitability of a source for generating heralded single photons.

### Entanglement demonstration

As mentioned above, different criteria can be exploited to demonstrate entanglement. In general, we can divide these into two classes:

1. those based on the violation of a Heisenberg-like inequality for the inferred variances, and
2. those based on the violation of Bell’s inequalities^80^.

For integrated sources, the vast majority of publications refer to the second class; thus, we focus on this. We refer the reader to the discussion related to Equation (C.1) in the Supplementary Section C, for further details on the first class.

Bell’s inequalities have been proposed as a condition that a quantum theory compatible with the local hidden-variables approach (as suggested by Einstein, Podolsky and Rosen^7^) must verify. The violation of Bell’s inequalities is not only a proof of entanglement but also demonstrates the non-local realism of quantum mechanics. For the maximally entangled states that are typically generated in SPDC and SFWM, a violation of Bell’s inequality can be exploited as proof of entanglement. We refer the reader to^81^ for a detailed description of the relation between entanglement and Bell’s inequalities.

A more ‘operative’ expression of Bell’s inequalities was proposed in 1969^82^; it relies on measuring the coincidence counts between the two arms (A and B) of a bi-partite entangled state for different detector settings. We consider the expression for polarisation entanglement (which is violated by entangled states)^83^:


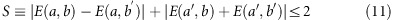


where *a*, *a’* and *b*, *b’* represent the settings for the two arms A and B (in this case, corresponding to the angles of the polarisers in front of the detectors), respectively, and


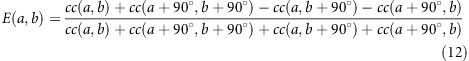


with *cc(a, b)* being the number of coincidences recorded with the signal and idler polarisers set to *a* and *b*, respectively. The angles that can lead to maximum violation of the CHSH (Clauser, Horne, Shimony, Holt) inequality for polarisation entangled states are *a*=0°, *a’*=45˚, *b*=22.5°, and *b’*=67.5˚.

A different kind of Bell’s inequality that can be exploited for demonstrating energy-time entanglement was described by Franson^84^. This state can be generated by pumping a nonlinear crystal with a CW pump having a coherence time larger than the coherence time of the down-converted photons. Energy-time entanglement is formally equivalent to polarisation entanglement when considering two time bins, where the horizontal and vertical polarisations are replaced by early (E) or late (L) time bins^85^ (thus the name time-bin entanglement). This two-mode energy-time entangled state can be generated by sending a pulsed laser through an unbalanced interferometer and then using the generated double-pulse as the pump for a SPDC or SFWM process^85^. With respect to polarisation entanglement, time-bin entanglement is more suitable for fibre propagation, as it is robust against polarisation fluctuations. Time-bin/Energy-time entanglement can be characterised by means of two unbalanced interferometers, one each for signal and idler photons, with variable phase shifters. A CHSH inequality similar to Equation (11) also holds in this case, with the angles of the polarisers substituted by the phase of the signal and idler interferometers. For the typical time-bin entangled state (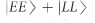), the maximal violation of the CHSH inequality is obtained for *a*=π/4, *b*=0, *a’*=-π/4, and *b’*=π/2^86^. Assuming the same average visibility, *V*, of the coincidence between the output ports of 4 interferometers (s1-i1, s1-i2, s2-i1, s2-i2), the CHSH inequality is violated when 
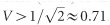
. See Supplementary Section C, for a discussion on the relationship between entanglement and non-classical correlations.

### Complex quantum state generation

While most research on the generation of quantum states addresses standard two-partite bi-dimensional states, such as polarisation entangled (2 dimensions) signal and idler pairs, the ability to generate more complex quantum states will strongly benefit applications in communications and computing. On the one hand, high-dimensional quantum states (so-called ‘quDits’) will increase the amount of information per single photon for quantum communications^55^. On the other hand, cluster states^87^, that is, multipartite entangled states in which each particle is entangled with more than one other particle, have been proposed as a fundamental tool for one-way quantum computing^88^. This novel form of computing relies on complex quantum states and simple measurements rather than a complex set of unitary operations on each qubit, as in the more standard circuit model for quantum computing. While cluster states and quDits have been generated in bulk-optic and free-space approaches (see, for example, Refs. 89, 90, 91 and Refs. 3, 54, 55, 56, 57), both remain an open challenge in chip form, although recent approaches have come close^92, 93^, and integrated sources of robust multipartite states based on SFWM have been theoretically predicted^94^.

## On-chip photon sources

In this section, we review recent advances in sources of single and entangled photons based on nonlinear processes taking place on an integrated chip. While the development of quantum sources using bulk optics is quite a mature field, a more widespread adoption of quantum technologies will require the miniaturisation of devices towards the chip level. This will reduce cost, footprint, and energy consumption and greatly increase reliability.

We classify these integrated sources according to whether they are based on waveguides or cavities, the latter often being used to enhance the nonlinearity as well as to provide the unique characteristics of the generated photons (such as narrow bandwidths). Table 1 compares state-of-the-art performances for single- and paired-photon sources for a range of structures, including microcavities, with a focus on CMOS-compatible integrated chips.

### Waveguides

Most integrated sources of quantum states of light are based on centrosymmetric materials such as silicon, silica (SiO_2_), silicon nitride (Si_3_N_4_), and silicon oxy-nitride (SiO_x_N_y_), which only have third-order nonlinearities^104^. However, there has also been substantial interest in noncentrosymmetric (or χ^(2)^) materials such as lithium niobate and III-V semiconductors. While possessing both a χ^(2)^ and χ^(3)^, they are referred to as ‘χ^(2)^’ materials since the second-order response dominates the χ^(3)^ response. We briefly discuss these platforms first.

While often requiring challenging fabrication processes, III-V semiconductors such as AlGaAs offer many advantages, including exhibiting a χ^(2)^ response and being a direct bandgap semiconductor that can provide optical gain via electrical pumping. One drawback, however, is that III-Vs lack birefringence; thus, phase matching requires novel techniques such as quasi-phase matching (QPM)^105, 106^ using, for example, Bragg grating reflection waveguides^107^ or quantum well intermixing^108^. Polarisation^109, 110, 111^, time-bin^112^ and energy-time^113^ entanglement have been achieved using these methods. Correlated photon pairs have also recently been generated in AlGaAs waveguides by exploiting their χ^(3)^ nonlinearity^114^.

Periodically poled lithium niobate (PPLN) QPM waveguides^115, 116^ have been used to successfully generate cross-polarised photon pairs^117, 118^ and polarisation entanglement via direct type II configurations^119^ by combining either two type II processes using two different poling periods^120, 121, 122^ or two type I processes by inserting a half-wave plate^123^. Time-bin entanglement^116, 124^, quantum state generation and manipulation^125, 126, 127^, ‘active’ quantum walks through nonlinear waveguide arrays^128, 129, 130^ and photon triplet generation^131^ have also all been demonstrated using this platform. By coating a PPLN waveguide with mirror-like facets, a monolithic OPO-based source of energy-time entangled photons^132^ has been demonstrated.

The generation of photon pairs in silicon waveguides was considered theoretically in 2006^133^ and demonstrated shortly after^134^. Time-bin^135^ and polarisation^136^ entangled photons were reported, initially with fibre components (Sagnac loop) and then in fully integrated form^99^, exploiting a monolithic polarisation rotator to combine two type 0 processes. Initially, pulsed pumps were used to achieve sufficient generation rates, but more recently, continuous wave (CW) pumping has been achieved^137^, and this is now common. The co-integration of silicon sources with silica devices such as arrayed waveguide gratings (AWGs) has been proven to be a powerful technique^138^.

### Microcavities and microresonators

Integrated optical cavities greatly enhance light-matter interaction by spatially or temporally confining and enhancing the radiation by several orders of magnitude, particularly with resonators having quality factors (*Q*=*ω/Δω*, where *ω* is the resonance frequency and *Δω* is the resonance width) of 10^6^ or even higher. For both highly nonlinear materials, such as silicon or III-V compounds, and more modestly nonlinear materials, such as Si_3_N_4_ and Hydex, cavities offer extreme enhancements in efficiency that can result in parametric fluorescence with pump powers on the order of microwatts only. Furthermore, given their small dimensions, cavities can readily be integrated on a chip with other photonic components.

Microdisc or microtoroid resonators confine light in whispering gallery modes and can achieve extremely high quality factors^139^. Silica microtoroids have achieved emission of photon pairs with CAR values >10^3^ and a spectral brightness surpassing that of PPLN bulk crystal sources^140^. Lithium niobate microtoroids have demonstrated the emission of squeezed light (twin beams) far above the OPO threshold^141^, as well as the emission of truly single-mode photon pairs^142^.

Photonic crystal (PhC) membrane waveguides, both in silicon and III-V semiconductors, are promising sources of non-classical states of light since they enable extreme light confinement that provides a strong enhancement of optical nonlinearities^143, 144, 145^. Line-defect, slow-light, PhC waveguides can reduce the group velocity of light to less than 1/50 of its natural speed while keeping the propagation losses low^146^. Correlated photon-pair generation via slow-light enhanced SFWM has been reported^147, 148, 149, 150^, as well as heralded photon-pair generation in III-V PhC waveguides^151^ and even high-dimensional time-bin entangled photons^102^. These experiments achieved a significant enhancement of pair generation efficiency with a strongly reduced footprint compared with conventional photon-pair sources.

Photonic crystal nanocavities <*λ*^3^ in size and with very high quality factors provide the ultimate interaction between light and matter^152, 153, 154^. Microwatt photon-pair generation via SFWM has been reported in a three PhC coupled cavity designed to yield triple resonances at the pump, signal and idler frequencies in an ultrasmall volume (< μm^3^)^155^. While fabrication challenges are significant, these nanocavities are promising, high-efficiency, ultralow power sources of quantum states of light. Recently, single-photon nonlinearities^156, 157^ were achieved in ultrahigh *Q/V* (quality-factor to volume ratio) nanocavities, with the future promise of integrated single-photon sources operating at room temperature via the photon-blockade effect^158, 159^.

In ring resonators, perhaps the most widely exploited microcavity in quantum photonics, the SFWM^160, 161^ efficiency for generating photon pairs using χ^(3)^ is ~γ *Q*^3^/*R*^2^ (where γ is the waveguide nonlinear parameter, *Q* is the quality factor and *R* is the radius^160^). This was experimentally verified for silicon rings with *R*=5-30 μm^162^ and highlights the trade-off between volume and Q factor. Ring resonators offer extremely high enhancement, particularly for a triply resonant cavity, which occurs if the total dispersion is low (that is, within a constant free spectral range, FSR=*v*_g_/(2π*R*), where *v*_g_ is the group velocity). Efficient dispersion engineering has been achieved in both silicon and SiN platforms^104^. Initial experiments verified the coincidences between signal and idler photons sent to different single-photon detectors by measuring the inter-beam *g*^(2)^ ^137^, in which generation rates of 10^5^ Hz with a CAR of 30 were achieved using <1 dBm CW pump power. A better figure of merit of 10^7^ Hz with a CAR of 50, achieved under the same pumping conditions, was later demonstrated in a 10 μm ring with a *Q* of 10^4^^162^.

Ring resonators are particularly promising sources of time-energy or time-bin entangled states in the telecom band for QKD applications^100, 101, 163^. Their narrow emission bandwidths, on the order of a few GHz, are compatible with DWDM (dense wavelength division multiplexing) networks, and the required frequency and low power of the pump makes remote pumping possible, with the resulting spectral brightness being comparable to the best second-order nonlinear crystals^100^. In addition, ultrahigh *Q* resonators yield extremely narrow linewidths, commensurate with quantum memories that typically rely on atomic transitions with linewidths on the order of 100 MHz or less^164^. CROW (coupled-resonator optical waveguide) devices increase the nonlinear parameter by ten times or more^165^ and have been shown to be efficient heralded single-photon sources^148^, wavelength multiplexed photon-pair sources^166^ and time-bin entangled photon^167^ sources.

Finally, it has been shown that ring resonators are particularly appealing for heralding single photons in a pure state without the need for external spectral filtering. In fact, when used as a heralded single-photon source, a typical resonator pumped by a field having a spectral width broader than the resonance linewidth can generate heralded single photons with a purity as high as 92%^18, 73, 160^. Moreover, it has been recently suggested that the individual control of the spectral width of the resonances involved in SFWM can lead to fully spectrally unentangled photon pairs; in this case, the purity can theoretically reach 100%^168^.

One challenge with SFWM – whether in waveguides or cavities – is that the pump exists in the same spectral region as the generated photon pairs instead of at twice their frequency, as in SPDC. This makes filtering out the pump, which is typically 90-100 dB stronger than the generated signal and idlers, a significant challenge. Very recently, however, this level of rejection was demonstrated on a chip^169^ for pair generation^170^.

Silicon has, in many ways, been the ‘workhorse’ for quantum applications based on integrated nanophotonics. The use of standard 45 nm CMOS fabrication processes has enabled the integration of ring resonators with electronic components^171^ as well as with other optical devices, such as filters, modulators, detectors, and splitters of degenerate photon pairs^172^. However, the moderately high linear (a few dB/cm) and significant nonlinear loss (two-photon absorption – TPA) of silicon have proven to be important limitations, despite the use of novel techniques such as integrating P-I-N junctions to sweep away TPA-generated free carriers. In turn, this allow higher pump powers to yield larger emission rates of 10^8^ Hz^77^.

**Figure 5:**
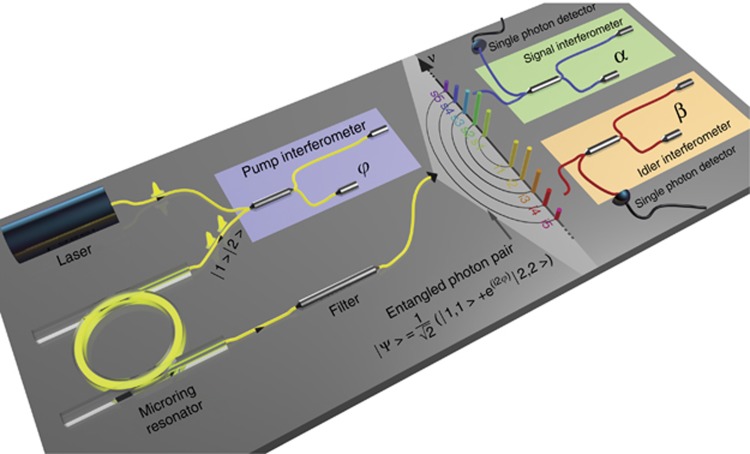
Quantum frequency comb generation and detection setup based on time-bin entanglement in a ring resonator^92^. A pulsed laser (16.8 MHz repetition rate passively mode-locked fibre laser with a bandwidth of 0.1 nm, spectrally centred at 1556.2 nm) is passed through an unbalanced Michelson interferometer (consisting of a 50/50 beam splitter, Faraday mirrors, and a phase shifter), generating two pulses with a phase difference *φ* in two respective time slots (time bins |1〉 and |2〉). The pulses are fed into the micro-ring resonator (see arrows for the propagation direction), exciting one micro-ring resonance. The nonlinear spontaneous four-wave mixing process generates signal-idler photon pairs on several ring resonances symmetric to the excited resonance (optical frequency comb, indicated in multicolour), either within the first or the second time slot (the generation in both time bins is made highly improbable by the chosen low excitation power). The superposition of the state generated in the first and the second time slot results in an entangled state output |ψ〉, which takes place simultaneously on several resonances and leads to a frequency comb of time-bin entangled photon pairs. For analysis purposes (entanglement verification or quantum state tomography), each photon of the spectrally filtered photon pair (distributed on two resonances symmetric to the excitation frequency, for example, the resonance pair i4-s4 used here) is individually passed through an interferometer, with the temporal imbalance equal to the time slot separation, and then detected using a single-photon detector (note that the phases *α* and *β* of the second and third interferometers can be individually controlled).

This has led to the need for developing new nonlinear platforms, including Si_3_N_4_ and Hydex^11^, that exhibit both extremely low linear and, perhaps more importantly, low nonlinear optical loss^173, 174^. Although Hydex – similar to silicon oxynitride – has a lower nonlinearity than silicon, very high Q ring resonators can be achieved (>10^6^), which greatly enhances the SFWM^98, 175, 176^. The emission of pairs for heralded single-photon sources was demonstrated over a 200 GHz multifrequency comb compatible with the ITU frequency grid for dense wavelength division multiplexed optical networks^97^. This would allow the transmission of quantum states over fibre-optic networks, as well as the use of standard telecom filters to route the different wavelengths and deterministically separate signal and idler photons. The high Q factor yielded photon pairs with narrow linewidths – compatible with quantum memories (~150 MHz). Very recently, the emission of entangled photons was also reported, with the multifrequency nature of the emitted signal idler pairs being exploited to enable an on-chip source of four-photon time-bin entangled states^92^ (Figure 5). In moderate refractive index materials such as Hydex, fibre-to-chip coupling can be extremely efficient; this coupling has allowed the use of self-pumping techniques with optical amplifiers to avoid the need for expensive external tuneable lasers, which is important for practical applications^97, 177^. Advanced time-bin entanglement circuits have also been reported in ultralow-loss silicon nitride photonic chips^178^. Recently, Hydex micro-ring resonators achieved type II SFWM on a chip by exploiting subtle birefringent effects, thus paving the way for the direct generation of polarisation entanglement on a chip in a single process^179^. Silicon nitride (Si_3_N_4_) ring resonators are also very interesting candidates as generators of quantum optical states^180^, including entangled photon pairs^103^, twin beams^181, 182^, and random numbers^183^.

## Deterministic sources

Deterministic photon sources are desired for many applications, such as quantum computing and communications, since the interaction probability between multiple single photons from independent random sources is far too low to be practical. While non-classical emitters such as quantum dots^184, 185, 186^ or nitrogen vacancies in diamonds^187^ can produce single photons deterministically and are promising sources, they are not without their challenges. Photon collection losses can degrade their deterministic nature, and even though photons created from the same emitter show very high indistinguishability^184, 185^, achieving enough uniformity with nanoscale accuracy^186, 187^ to generate indistinguishable photons from multiple emitters is difficult, often requiring narrowband filtering^186^.

Photon generation via nonlinear optics also has its challenges, as it is intrinsically random, being governed by statistical distributions (for example, Poissonian and thermal) that limit the single-photon generation probability to less than 25%^188^. However, ‘heralding’ can increase the probability of single-photon generation without sacrificing the source quality through the use of, for example, active multiplexing techniques^150, 189, 190, 191, 192, 193, 194, 195^. More importantly, photons from separate nonlinear sources have been shown to be highly indistinguishable^195^.

**Figure 6:**
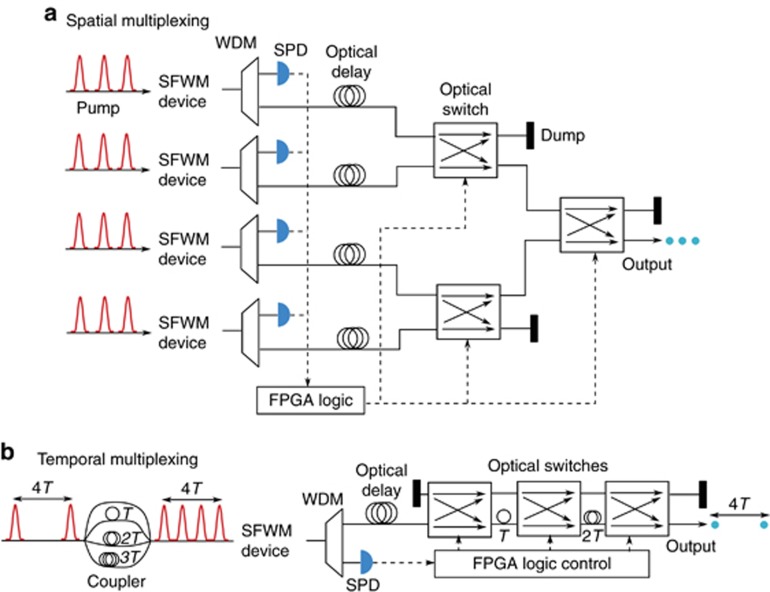
Schemes for spatial (**a**) and temporal (**b**) multiplexing. WDM: wavelength division multiplexing, SPD: single-photon detector, FPGA: field programmable gate array.

Photon multiplexing can be achieved in space^150, 189, 190^ or time^191, 192, 193, 194, 195^. Figure 6 shows two multiplexing schemes that can actively combine heralded photons from *N* different modes (in this case, *N*=4). In spatial multiplexing, as shown in Figure 6a, correlated photon pairs are randomly generated in some of the waveguides via SFWM. One and only one heralded photon at a time is routed to the output according to predefined logic in a field-programmable gate array (FPGA); thus, the single-photon output probability is enhanced^150^. This scheme, however, requires many devices for each photon source and thus is difficult to scale up. Temporal multiplexing, as illustrated in Figure 6b, is much more efficient because only one photon source is required and the photons to be multiplexed are generated from different temporal modes. When photons from 4 modes are multiplexed, the enhancement of the single-photon output probability is 100%, and the Hong-Ou-Mandel (HOM^196^) interference with the multiplexed photons exhibits 91% visibility^195^. So far, however, the single-photon generation efficiency after multiplexing has been very low. This is mainly because the starting point for multiplexing – the source before multiplexing – has to operate in the low efficiency regime to avoid multiphoton noise. If photon-number-resolving detectors^197^ can be exploited, one can start at the theoretical limit of 25% single-photon generation probability and use scalable temporal multiplexing schemes to achieve nearly deterministic single-photon sources. Of course, the overall loss, including, in particular, the loss due to the switches^195^, is a critical factor since this can significantly degrade the overall fidelity of a single-photon source.

## Conclusions

We review the current state-of-the-art in photonic integrated circuits designed to generate complex photonic quantum states, focusing on devices based on nonlinear optics that are compatible with quantum memories, with fibre optic communications, as well as with silicon integrated circuit semiconductor technology (CMOS). These new developments play a key role in realising compact, low-cost, and practical sources of complex quantum optical states on a chip, which will ultimately enable quantum technologies to have a significant impact on our society.

## Figures and Tables

**Table 1:** Summary of typical experimental results in various χ^(3)^ structures

Structures	Silicon			Hydex	Si_3_N_4_
Parameters	Nanowire^95^	Ring^77^	PhC^96^	Ring^97^	Ring
Nonlinear coefficient (W^−1^m^−1^)	300	-	4000	0.22^98^	-
Q-factor	-	37500	-	1375000	2000000
Coupled pump average power (mW)	0.18	0.019	0.055	21	3
Collected photon bandwidth (GHz)	25	5.2	50	0.11	0.09
Brightness (pairs s^-1^ mW^-2^ GHz^-1^)	1.6 × 10^5^	4.4 × 10^8^	1.5 × 10^6^	6.2 × 10^3^	4.3 × 10^8^
CAR	320	602	330	11	-
*g*^(2)^(0)	-	-	0.09	0.14	-
Number of entangled photons	2^99^	2^100, 101^	2^102^	4^92^	2^103^

## References

[bib1] O’Brien JL. Optical quantum computing. Science 2007; 318: 1567–1570. 18063781 10.1126/science.1142892

[bib2] Giovannetti V, Lloyd S, Maccone L. Quantum-enhanced measurements: beating the standard quantum limit. Science 2004; 306: 1330–1336. 15550661 10.1126/science.1104149

[bib3] Gisin N, Ribordy G, Tittel W, Zbinden H. Quantum cryptography. Rev Mod Phys 2002; 74: 145–195.

[bib4] Bertlmann RA, Zeilinger A. Quantum (Un)speakables: From Bell to Quantum Information. Berlin Heidelberg: Springer; 2002, 119–153.

[bib5] Giustina M, Versteegh MAM, Wengerowsky S, Handsteiner J, Hochrainer A et al. Significant-Loophole-Free Test of Bell’s Theorem with Entangled Photons. Phys Rev Lett 2015; 115: 250401. 26722905 10.1103/PhysRevLett.115.250401

[bib6] Shalm LK, Meyer-Scott E, Christensen BG, Bierhorst P, Wayne MA et al. Strong Loophole-Free Test of Local Realism. Phys Rev Lett 2015; 115: 250402. 26722906 10.1103/PhysRevLett.115.250402 PMC5815856

[bib7] Einstein A, Podolsky B, Rosen N. Can Quantum-Mechanical Description of Physical Reality Be Considered Complete? Phys Rev 1935; 47: 777–780.

[bib8] Della Valle G, Osellame R, Laporta P. Micromachining of photonic devices by femtosecond laser pulses. J Opt A Pure Appl Opt 2009; 11: 13001.

[bib9] O’Brien J, Patton B, Sasaki M, Vučković J. Focus on integrated quantum optics. New J Phys 2013; 15: 035016.

[bib10] Thomas-Peter N, Langford NK, Datta A, Zhang L, Smith BJ et al. Integrated photonic sensing. New J Phys 2011; 13: 055024.

[bib11] Takesue H, Inoue K. Generation of 1.5-μm band time-bin entanglement using spontaneous fiber four-wave mixing and planar light-wave circuit interferometers. Phys Rev A 2005; 72: 041804.

[bib12] Politi A, Cryan MJ, Rarity JG, Yu SY, O’Brien JL. Silica-on-Silicon Waveguide Quantum Circuits. Science 2008; 320: 646–649. 18369104 10.1126/science.1155441

[bib13] Marshall GD, Politi A, Matthews JCF, Dekker P, Ams M et al. Laser written waveguide photonic quantum circuits. Opt Express 2009; 17: 12546–12554. 19654657 10.1364/oe.17.012546

[bib14] Meany T, Gräfe M, Heilmann R, Perez-Leija A, Gross S et al. Laser written circuits for quantum photonics. Laser Photonics Rev 2015; 9: 363–384.

[bib15] Matthews JCF, Politi A, Stefanov A, O’Brien JL. Manipulation of multiphoton entanglement in waveguide quantum circuits. Nat Photonics 2009; 3: 346–350.

[bib16] Smith BJ, Kundys D, Thomas-Peter N, Smith PGR, Walmsley IA. Phase-controlled integrated photonic quantum circuits. Opt Express 2009; 17: 13516–13525. 19654759 10.1364/oe.17.013516

[bib17] Bonneau D, Engin E, Ohira K, Suzuki N, Yoshida H et al. Quantum interference and manipulation of entanglement in silicon wire waveguide quantum circuits. New J Phys 2012; 14: 045003.

[bib18] Silverstone JW, Bonneau D, Ohira K, Suzuki N, Yoshida H et al. On-chip quantum interference between silicon photon-pair sources. Nat Photonics 2013; 8: 104–108.

[bib19] Metcalf BJ, Thomas-Peter N, Spring JB, Kundys D, Broome MA et al. Multiphoton quantum interference in a multiport integrated photonic device. Nat Commun 2013; 4: 1356. 23322044 10.1038/ncomms2349

[bib20] Preble SF, Fanto ML, Steidle JA, Tison CC, Howland GA et al. On-Chip Quantum Interference from a Single Silicon Ring-Resonator Source. Phys Rev Appl 2015; 4: 021001.

[bib21] Silverstone JW, Santagati R, Bonneau D, Strain MJ, Sorel M et al. Qubit entanglement between ring-resonator photon-pair sources on a silicon chip. Nat Commun 2015; 6: 7948. 26245267 10.1038/ncomms8948 PMC4918336

[bib22] Laing A, Peruzzo A, Politi A, Verde MR, Halder M et al. High-fidelity operation of quantum photonic circuits. Appl Phys Lett 2010; 97: 211109.

[bib23] Crespi A, Ramponi R, Osellame R, Sansoni L, Bongioanni I et al. Integrated photonic quantum gates for polarization qubits. Nat Commun 2011; 2: 566. 22127062 10.1038/ncomms1570 PMC3482629

[bib24] Politi A, Matthews JCF, O’Brien JL. Shor’s quantum factoring algorithm on a photonic chip. Science 2009; 325: 1221. 19729649 10.1126/science.1173731

[bib25] Perets HB, Lahini Y, Pozzi F, Sorel M, Morandotti R et al. Realization of quantum walks with negligible decoherence in waveguide lattices. Phys Rev Lett 2008; 100: 170506. 18518267 10.1103/PhysRevLett.100.170506

[bib26] Peruzzo A, Lobino M, Matthews JCF, Matsuda N, Politi A et al. Quantum walks of correlated photons. Science 2010; 329: 1500–1503. 20847264 10.1126/science.1193515

[bib27] Owens JO, Broome MA, Biggerstaff DN, Goggin ME, Fedrizzi A et al. Two-photon quantum walks in an elliptical direct-write waveguide array. New J Phys 2011; 13: 075003.

[bib28] Sansoni L, Sciarrino F, Vallone G, Mataloni P, Crespi A et al. Two-Particle Bosonic-Fermionic Quantum Walk via Integrated Photonics. Phys Rev Lett 2012; 108: 010502. 22304249 10.1103/PhysRevLett.108.010502

[bib29] Poulios K, Keil R, Fry D, Meinecke JDA, Matthews JCF et al. Quantum Walks of Correlated Photon Pairs in Two-Dimensional Waveguide Arrays. Phys Rev Lett 2014; 112: 143604. 24765962 10.1103/PhysRevLett.112.143604

[bib30] Broome MA, Fedrizzi A, Rahimi-Keshari S, Dove J, Aaronson S et al. Photonic Boson Sampling in a Tunable Circuit. Science 2013; 339: 794–798. 23258411 10.1126/science.1231440

[bib31] Spring JB, Metcalf BJ, Humphreys PC, Kolthammer WS, Jin XM et al. Boson sampling on a photonic chip. Science 2013; 339: 798–801. 23258407 10.1126/science.1231692

[bib32] Tillmann M, Dakić B, Heilmann R, Nolte S, Szameit A et al. Experimental boson sampling. Nat Photonics 2013; 7: 540–544.

[bib33] Crespi A, Osellame R, Ramponi R, Brod DJ, Galvão EF et al. Integrated multimode interferometers with arbitrary designs for photonic boson sampling. Nat Photonics 2013; 7: 545–549.

[bib34] Ralph TC. Quantum computation: Boson sampling on a chip. Nat Photonics 2013; 7: 514–515.

[bib35] Giovannetti V, Lloyd S, Maccone L. Advances in quantum metrology. Nat Photonics 2011; 5: 222–229.

[bib36] Ladd TD, Jelezko F, Laflamme R, Nakamura Y, Monroe C et al. Quantum computers. Nature 2010; 464: 45–53. 20203602 10.1038/nature08812

[bib37] Natarajan CM, Tanner MG, Hadfield RH. Superconducting nanowire single-photon detectors: physics and applications. Supercond Sci Technol 2012; 25: 063001.

[bib38] Chunnilall CJ, Degiovanni IP, Kück S, Müller I, Sinclair AG. Metrology of single-photon sources and detectors: a review. Opt Eng 2014; 53: 081910.

[bib39] Reithmaier G, Lichtmannecker S, Reichert T, Hasch P, Müller K et al. On-chip time resolved detection of quantum dot emission using integrated superconducting single photon detectors. Sci Rep 2013; 3: 1901. 23712624 10.1038/srep01901 PMC3664895

[bib40] Li J, Kirkwood RA, Baker LJ, Bosworth D, Erotokritou K et al. Nano-optical single-photon response mapping of waveguide integrated molybdenum silicide (MoSi) superconducting nanowires. Opt Express 2016; 24: 13931–13938. 27410555 10.1364/OE.24.013931

[bib41] Rath P, Kahl O, Ferrari S, Sproll F, Lewes-Malandrakis G et al. Superconducting single-photon detectors integrated with diamond nanophotonic circuits. Light Sci Appl 2015; 4: e338.

[bib42] Kahl O, Ferrari S, Kovalyuk V, Goltsman GN, Korneev A. Pernice WHP. Waveguide integrated superconducting single-photon detectors with high internal quantum efficiency at telecom wavelengths. Sci Rep 2015; 5: 10941. 26061283 10.1038/srep10941 PMC4462017

[bib43] Matsuda N, Takesue H. Generation and manipulation of entangled photons on silicon chips. Nanophotonics 2016; 5: 440–455.

[bib44] Masada G, Furusawa A. On-chip continuous-variable quantum entanglement. Nanophotonics 2016; 5: 469–482.

[bib45] Xiong CL, Bell B, Eggleton BJ. CMOS-compatible photonic devices for single-photon generation. Nanophotonics 2016; 5: 427–439.

[bib46] Caspani L, Reimer C, Kues M, Roztocki P, Clerici M et al. Multifrequency sources of quantum correlated photon pairs on-chip: a path toward integrated Quantum Frequency Combs. Nanophotonics 2016; 5: 351–362.

[bib47] Orieux A, Diamanti E. Recent advances on integrated quantum communications. J Opt 2016; 18: 083002.

[bib48] Savanier M, Kumar R, Mookherjea S. Photon pair generation from compact silicon microring resonators using microwatt-level pump powers. Opt Express 2016; 24: 3313–3328. 26906993 10.1364/OE.24.003313

[bib49] Bonneau D, Silverstone JW, Thompson MGSilicon Quantum Photonics. In: Pavesi L, Lockwood DJ editors. Silicon Photonics III. Berlin Heidelberg: Springer; 2016, 41–82.

[bib50] Hendrickson SM, Foster AC, Camacho RM, Clader BD. Integrated nonlinear photonics: emerging applications and ongoing challenges [Invited]. J Opt Soc Am B 2014; 31: 3193–3203.

[bib51] Tanzilli S, Martin A, Kaiser F, De Micheli MP, Alibart O et al. On the genesis and evolution of integrated quantum optics. Laser Photonics Rev 2012; 6: 115–143.

[bib52] Benenti G, Casati G, Strini G. Principles of quantum computation and information Volume I: Basic concepts. Singapore: World Scientific; 2004.

[bib53] Ekert AK. Quantum cryptography based on Bell’s theorem. Phys Rev Lett 1991; 67: 661–663. 10044956 10.1103/PhysRevLett.67.661

[bib54] Kolobov MI. The spatial behavior of nonclassical light. Rev Mod Phys 1999; 71: 1539–1589.

[bib55] Ali-Khan I, Broadbent CJ, Howell JC. Large-alphabet quantum key distribution using energy-time entangled bipartite states. Phys Rev Lett 2007; 98: 060503. 17358925 10.1103/PhysRevLett.98.060503

[bib56] Mower J, Zhang ZS, Desjardins P, Lee C, Shapiro JH et al. High-dimensional quantum key distribution using dispersive optics. Phys Rev A 2013; 87: 062322.

[bib57] Mair A, Vaziri A, Weihs G, Zeilinger A. Entanglement of the orbital angular momentum states of photons. Nature 2001; 412: 313–316. 11460157 10.1038/35085529

[bib58] Bennett CH, Brassard GQuantum cryptography: public key distribution and coin tossingProceeding of IEEE International Conference on Computers, Systems and Signal Processing; Bangalore, India. Banglore, India: IEEE; 1984, 175–179.

[bib59] Wootters WK, Zurek WH. A single quantum cannot be cloned. Nature 1982; 299: 802–803.

[bib60] Dieks D. Communication by EPR devices. Phys Lett A 1982; 92: 271–272.

[bib61] Knill E, Laflamme R, Milburn GJ. A scheme for efficient quantum computation with linear optics. Nature 2001; 409: 46–52. 11343107 10.1038/35051009

[bib62] Loudon R. The Quantum Theory of Light. 3rd edn. Oxford: Oxford University Press; 2000.

[bib63] Villar AS, Cruz LS, Cassemiro KN, Martinelli M, Nussenzveig P. Generation of Bright Two-Color Continuous Variable Entanglement. Phys Rev Lett 2005; 95: 243603. 16384378 10.1103/PhysRevLett.95.243603

[bib64] Mandel L, Wolf E. Optical Coherence and Quantum Optics. Cambridge: Cambridge University Press; 1995.

[bib65] Kok P, Munro WJ, Nemoto K, Ralph TC, Dowling J et al. Linear optical quantum computing with photonic qubits. Rev Mod Phys 2007; 79: 135–174.

[bib66] Ekert A. Entangled quantum systems and the Schmidt decomposition. Am J Phys 1995; 63: 415–423.

[bib67] Leonhardt U. Measuring the quantum state of light. Cambridge: Cambridge University Press; 1997.

[bib68] Bachor HA, Ralph TC. A guide to experiments in quantum optics. 2nd edn. Wiley-VCH: Berlin; 2004.

[bib69] James DFV, Kwiat PG, Munro WJ, White AG. Measurement of qubits. Phys Rev A 2001; 64: 052312.

[bib70] Mosley PJ, Lundeen JS, Smith BJ, Wasylczyk P, U’Ren AB et al. Heralded Generation of Ultrafast Single Photons in Pure Quantum States. Phys Rev Lett 2008; 100: 133601. 18517952 10.1103/PhysRevLett.100.133601

[bib71] Migdall A, Polyakov SV, Fan JY, Bienfang JC. Single-photon generation and detection physics and applications. Burlington: Academic Press; 2013.

[bib72] Fang B, Cohen O, Liscidini M, Sipe JE, Lorenz VO. Fast and highly resolved capture of the joint spectral density of photon pairs. Optica 2014; 1: 281–284.

[bib73] Grassani D, Simbula A, Pirotta S, Galli M, Menotti M et al. Energy correlations of photon pairs generated by a silicon microring resonator probed by Stimulated Four Wave Mixing. Sci Rep 2016; 6: 23564. 27032688 10.1038/srep23564 PMC4817032

[bib74] Mandel L. Fluctuations of Photon Beams and their Correlations. Proc Phys Soc 1958; 72: 1037–1048.

[bib75] Wolf E. Correlation between Photons in Partially Polarized Light Beams. Proc Phys Soc 1960; 76: 424–426.

[bib76] Pomarico E, Sanguinetti B, Guerreiro T, Thew R, Zbinden H. MHz rate and efficient synchronous heralding of single photons at telecom wavelengths. Opt Express 2012; 20: 23846–23855. 23188350 10.1364/OE.20.023846

[bib77] Engin E, Bonneau D, Natarajan CM, Clark AS, Tanner MG et al. Photon pair generation in a silicon micro-ring resonator with reverse bias enhancement. Opt Express 2013; 21: 27826–27834. 24514299 10.1364/OE.21.027826

[bib78] Vernon Z, Liscidini M, Sipe JE. No free lunch: the trade-off between heralding rate and efficiency in microresonator-based heralded single photon sources. Opt Lett 2016; 41: 788–791. 26872189 10.1364/ol.41.000788

[bib79] Takesue H, Shimizu K. Effects of multiple pairs on visibility measurements of entangled photons generated by spontaneous parametric processes. Opt Commun 2010; 283: 276–287.

[bib80] Bell JS. On the Einstein-Podolsky-Rosen Paradox. Physics 1964; 1: 195–200.

[bib81] Munro WJ, Nemoto K, White AG. The Bell inequality: a measure of entanglement? J Mod Opt 2001; 48: 1239–1246.

[bib82] Clauser JF, Horne MA, Shimony A, Holt RA. Proposed Experiment to Test Local Hidden-Variable Theories. Phys Rev Lett 1969; 23: 880–884.

[bib83] Shimony ABell’s Theorem. In: Zalta EN editor. The Stanford Encyclopedia of Philosophy. Palo Alto: Stanford University; 2016.

[bib84] Franson JD. Bell inequality for position and time. Phys Rev Lett 1989; 62: 2205–2208. 10039885 10.1103/PhysRevLett.62.2205

[bib85] Brendel J, Gisin N, Tittel W, Zbinden H. Pulsed Energy-Time Entangled Twin-Photon Source for Quantum Communication. Phys Rev Lett 1999; 82: 2594–2597.

[bib86] Lima G, Vallone G, Chiuri A, Cabello A, Mataloni P. Experimental Bell-inequality violation without the postselection loophole. Phys Rev A 2010; 81: 040101.

[bib87] Briegel HJ, Raussendorf R. Persistent Entanglement in Arrays of Interacting Particles. Phys Rev Lett 2001; 86: 910–913. 11177971 10.1103/PhysRevLett.86.910

[bib88] Raussendorf R, Briegel HJ. A one-way quantum computer. Phys Rev Lett 2001; 86: 5188–5191. 11384453 10.1103/PhysRevLett.86.5188

[bib89] Walther P, Resch KJ, Rudolph T, Schenck E, Weinfurter H et al. Experimental one-way quantum computing. Nature 2005; 434: 169–176. 15758991 10.1038/nature03347

[bib90] Pysher M, Miwa Y, Shahrokhshahi R, Bloomer R, Pfister O. Parallel generation of quadripartite cluster entanglement in the optical frequency comb. Phys Rev Lett 2011; 107: 030505. 21838341 10.1103/PhysRevLett.107.030505

[bib91] Pinel O, Jian P, de Araújo RM, Feng JX, Chalopin B et al. Generation and characterization of multimode quantum frequency combs. Phys Rev Lett 2012; 108: 083601. 22463528 10.1103/PhysRevLett.108.083601

[bib92] Reimer C, Kues M, Roztocki P, Wetzel B, Grazioso F et al. Generation of multiphoton entangled quantum states by means of integrated frequency combs. Science 2016; 351: 1176–1180. 26965623 10.1126/science.aad8532

[bib93] Ciampini MA, Orieux A, Paesani S, Sciarrino F, Corrielli G, Crespi A et al. Path-polarization hyperentangled and cluster states of photons on a chip. Light Sci Appl 2016; 5: e16064. 10.1038/lsa.2016.64 PMC6059950 30167159

[bib94] Menotti M, Maccone L, Sipe JE, Liscidini M. Generation of energy-entangled W states via parametric fluorescence in integrated devices. Phys Rev A 2016; 94: 013845.

[bib95] Harada KI, Takesue H, Fukuda H, Tsuchizawa T, Watanabe T et al. Frequency and Polarization Characteristics of Correlated Photon-Pair Generation Using a Silicon Wire Waveguide. IEEE J Sel Top Quantum Electron 2010; 16: 325–331.

[bib96] Xiong CL, Collins MJ, Steel MJ, Krauss TF, Eggleton BJ et al. Photonic Crystal Waveguide Sources of Photons for Quantum Communication Applications. IEEE J Sel Top Quantum Electron 2015; 21: 205–214.

[bib97] Reimer C, Caspani L, Clerici M, Ferrera M, Kues M et al. Integrated frequency comb source of heralded single photons. Opt Express 2014; 22: 6535–6546. 24664002 10.1364/OE.22.006535

[bib98] Ferrera M, Razzari L, Duchesne D, Morandotti R, Yang Z et al. Low-power continuous-wave nonlinear optics in doped silica glass integrated waveguide structures. Nat Photonics 2008; 2: 737–740.

[bib99] Matsuda N, Le Jeannic H, Fukuda H, Tsuchizawa T, Munro WJ, Shimizu K et al. A monolithically integrated polarization entangled photon pair source on a silicon chip. Sci Rep 2012; 2: 817. 23150781 10.1038/srep00817 PMC3495342

[bib100] Grassani D, Azzini S, Liscidini M, Galli M, Strain MJ et al. Micrometer-scale integrated silicon source of time-energy entangled photons. Optica 2015; 2: 88–94.

[bib101] Wakabayashi R, Fujiwara M, Yoshino K, Nambu Y, Sasaki M et al. Time-bin entangled photon pair generation from Si micro-ring resonator. Opt Express 2015; 23: 1103–1113. 25835870 10.1364/OE.23.001103

[bib102] Takesue H, Matsuda N, Kuramochi E, Notomi M. Entangled photons from on-chip slow light. Sci Rep 2014; 4: 3913. 24468821 10.1038/srep03913 PMC3904141

[bib103] Ramelow S, Farsi A, Clemmen S, Orquiza D, Luke K et al. Silicon-Nitride Platform for Narrowband Entangled Photon Generation. ArXiv 2015; 1508: 04358.

[bib104] Moss DJ, Morandotti R, Gaeta AL, Lipson M. New CMOS-compatible platforms based on silicon nitride and Hydex for nonlinear optics. Nat Photonics 2013; 7: 597–607.

[bib105] Helmy AS, Abolghasem P, Stewart Aitchison J, Bijlani BJ, Han J et al. Recent advances in phase matching of second-order nonlinearities in monolithic semiconductor waveguides. Laser Photonics Rev 2011; 5: 272–286.

[bib106] Lanco L, Ducci S, Likforman J-P, Marcadet X, van Houwelingen JAW et al. Semiconductor Waveguide Source of Counterpropagating Twin Photons. Phys Rev Lett 2006; 97: 173901. 17155475 10.1103/PhysRevLett.97.173901

[bib107] Horn R, Abolghasem P, Bijlani BJ, Kang DP, Helmy AS et al. Monolithic source of photon pairs. Phys Rev Lett 2012; 108: 153605. 22587254 10.1103/PhysRevLett.108.153605

[bib108] Sarrafi P, Zhu EY, Dolgaleva K, Holmes BM, Hutchings DC et al. Continuous-wave quasi-phase-matched waveguide correlated photon pair source on a III–V chip. Appl Phys Lett 2013; 103: 251115.

[bib109] Horn RT, Kolenderski P, Kang DP, Abolghasem P, Scarcella C et al. Inherent polarization entanglement generated from a monolithic semiconductor chip. Sci Rep 2013; 3: 2314. 23896982 10.1038/srep02314 PMC3727056

[bib110] Orieux A, Eckstein A, Lemaître A, Filloux P, Favero I et al. Direct Bell States Generation on a III-V Semiconductor Chip at Room Temperature. Phys Rev Lett 2013; 110: 160502. 23679588 10.1103/PhysRevLett.110.160502

[bib111] Vallés A, Hendrych M, Svozilík J, Machulka R, Abolghasem P et al. Generation of polarization-entangled photon pairs in a Bragg reflection waveguide. Opt Express 2013; 21: 10841–10849. 23669941 10.1364/OE.21.010841

[bib112] Sarrafi P, Zhu EY, Holmes BM, Hutchings DC, Aitchison S. High-visibility two-photon interference of frequency–time entangled photons generated in a quasi-phase-matched AlGaAs waveguide. Opt Lett 2014; 39: 5188–5191. 25166106 10.1364/OL.39.005188

[bib113] Autebert C, Bruno N, Martin A, Lemaitre A, Carbonell CG et al. Integrated AlGaAs source of highly indistinguishable and energy-time entangled photons. Optica 2016; 3: 143–146.

[bib114] Kultavewuti P, Zhu EY, Qian L, Pusino V, Sorel M et al. Correlated photon pair generation in AlGaAs nanowaveguides via spontaneous four-wave mixing. Opt Express 2016; 24: 3365–3376. 26906995 10.1364/OE.24.003365

[bib115] Tanzilli S, De Riedmatten H, Tittel W, Zbinden H, Baldi P et al. Highly efficient photon-pair source using periodically poled lithium niobate waveguide. Electron Lett 2001; 37: 26–28.

[bib116] Tanzilli S, Tittel W, De Riedmatten H, Zbinden H, Baldi P, DeMicheli M et al. PPLN waveguide for quantum communication. Eur Phys J D 2002; 18: 155–160.

[bib117] Nosaka T, Das BK, Fujimura M, Suhara T. Cross-polarized twin photon generation device using quasi-phase matched LiNbO/sub 3/ waveguide. IEEE Photonics Technol Lett 2006; 18: 124–126.

[bib118] Fujii G, Namekata N, Motoya M, Kurimura S, Inoue S. Bright narrowband source of photon pairs at optical telecommunication wavelengths using a type-II periodically poled lithium niobate waveguide. Opt Express 2007; 15: 12769–12776. 19550546 10.1364/oe.15.012769

[bib119] Suhara T, Okabe H, Fujimura M. Generation of Polarization-Entangled Photons by Type-II Quasi-Phase-Matched Waveguide Nonlinear-Optic Device. IEEE Photonics Technol Lett 2007; 19: 1093–1095.

[bib120] Suhara T, Nakaya G, Kawashima J, Fujimura M. Quasi-Phase-Matched Waveguide Devices for Generation of Postselection-Free Polarization-Entangled Twin Photons. IEEE Photonics Technol Lett 2009; 21: 1096–1098.

[bib121] Thyagarajan K, Lugani J, Ghosh S, Sinha K, Martin A et al. Generation of polarization-entangled photons using type-II doubly periodically poled lithium niobate waveguides. Phys Rev A 2009; 80: 052321.

[bib122] Herrmann H, Yang X, Thomas A, Poppe A, Sohler W et al. Post-selection free, integrated optical source of non-degenerate, polarization entangled photon pairs. Opt Express 2013; 21: 27981–27991. 24514311 10.1364/OE.21.027981

[bib123] Kawashima J, Fujimura M, Suhara T. Type-I Quasi-Phase-Matched Waveguide Device for Polarization-Entangled Twin Photon Generation. IEEE Photonics Technol Lett 2009; 21: 566–568.

[bib124] Honjo T, Takesue H, Inoue K. Generation of energy-time entangled photon pairs in 1.5-μm band with periodically poled lithium niobate waveguide. Opt Express 2007; 15: 1679–1683. 19532403 10.1364/oe.15.001679

[bib125] Bonneau D, Lobino M, Jiang PS, Natarajan CM, Tanner MG et al. Fast Path and Polarization Manipulation of Telecom Wavelength Single Photons in Lithium Niobate Waveguide Devices. Phys Rev Lett 2012; 108: 053601. 22400933 10.1103/PhysRevLett.108.053601

[bib126] Martin A, Alibart O, De Micheli MP, Ostrowsky DB, Tanzilli S. A quantum relay chip based on telecommunication integrated optics technology. New J Phys 2012; 14: 025002.

[bib127] Jin H, Liu FM, Xu P, Xia JL, Zhong ML et al. On-Chip Generation and Manipulation of Entangled Photons Based on Reconfigurable Lithium-Niobate Waveguide Circuits. Phys Rev Lett 2014; 113: 103601. 25238358 10.1103/PhysRevLett.113.103601

[bib128] Solntsev AS, Sukhorukov AA, Neshev DN, Kivshar YS. Spontaneous Parametric Down-Conversion and Quantum Walks in Arrays of Quadratic Nonlinear Waveguides. Phys Rev Lett 2012; 108: 023601. 22324683 10.1103/PhysRevLett.108.023601

[bib129] Hamilton CS, Kruse R, Sansoni L, Silberhorn C, Jex I. Driven Quantum Walks. Phys Rev Lett 2014; 113: 083602. 25192097 10.1103/PhysRevLett.113.083602

[bib130] Solntsev AS, Setzpfandt F, Clark AS, Wu CW, Collins MJ et al. Generation of nonclassical biphoton states through cascaded quantum walks on a nonlinear chip. Phys Rev X 2014; 4: 031007.

[bib131] Krapick S, Brecht B, Herrmann H, Quiring V, Silberhorn C. On-chip generation of photon-triplet states. Opt Express 2016; 24: 2836–2849. 26906852 10.1364/OE.24.002836

[bib132] Pomarico E, Sanguinetti B, Gisin N, Thew R, Zbinden H et al. Waveguide-based OPO source of entangled photon pairs. New J Phys 2009; 11: 113042.

[bib133] Lin Q, Agrawal GP. Silicon waveguides for creating quantum-correlated photon pairs. Opt Lett 2006; 31: 3140–3142. 17041661 10.1364/ol.31.003140

[bib134] Sharping JE, Lee KF, Foster MA, Turner AC, Schmidt BS et al. Generation of correlated photons in nanoscale silicon waveguides. Opt Express 2006; 14: 12388–12393. 19529670 10.1364/oe.14.012388

[bib135] Takesue H, Tokura Y, Fukuda H, Tsuchizawa T, Watanabe T et al. Entanglement generation using silicon wire waveguide. Appl Phys Lett 2007; 91: 201108.

[bib136] Takesue H, Fukuda H, Tsuchizawa T, Watanabe T, Yamada K et al. Generation of polarization entangled photon pairs using silicon wire waveguide. Opt Express 2008; 16: 5721–5727. 18542680 10.1364/oe.16.005721

[bib137] Clemmen S, Phan Huy K, Bogaerts W, Baets RG, Emplit P et al. Continuous wave photon pair generation in silicon-on-insulator waveguides and ring resonators. Opt Express 2009; 17: 16558–16570. 19770871 10.1364/OE.17.016558

[bib138] Matsuda N, Karkus P, Nishi H, Tsuchizawa T, Munro WJ et al. On-chip generation and demultiplexing of quantum correlated photons using a silicon-silica monolithic photonic integration platform. Opt Express 2014; 22: 22831–22840. 25321753 10.1364/OE.22.022831

[bib139] Armani DK, Kippenberg TJ, Spillane SM, Vahala KJ. Ultra-high-Q toroid microcavity on a chip. Nature 2003; 421: 925–928. 12606995 10.1038/nature01371

[bib140] Jiang WC, Lu XY, Zhang JD, Painter O, Lin Q. Silicon-chip source of bright photon pairs. Opt Express 2015; 23: 20884–20904. 26367942 10.1364/OE.23.020884

[bib141] Fürst JU, Strekalov DV, Elser D, Aiello A, Andersen UL et al. Quantum Light from a Whispering-Gallery-Mode Disk Resonator. Phys Rev Lett 2011; 106: 113901. 21469862 10.1103/PhysRevLett.106.113901

[bib142] Förtsch M, Schunk G, Fürst JU, Strekalov D, Gerrits T, Stevens MJ et al. Highly efficient generation of single-mode photon pairs from a crystalline whispering-gallery-mode resonator source. Phys Rev A 2015; 91: 023812.

[bib143] Joannopoulos JD, Johnson SG, Winn JN, Meade RD. Photonic Crystals Molding the Flow of Light. 2nd edn. Princeton: Princeton University Press; 2008.

[bib144] Krauss TF, De La Rue RM, Brand S. Two-dimensional photonic-bandgap structures operating at near-infrared wavelengths. Nature 1996; 383: 699–702.

[bib145] Notomi M. Manipulating light with strongly modulated photonic crystals. Reports Prog Phys 2010; 73: 096501.

[bib146] O’Faolain L, Schulz SA, Beggs DM, White TP, Spasenović M et al. Loss engineered slow light waveguides. Opt Express 2010; 18: 27627–27638. 21197037 10.1364/OE.18.027627

[bib147] Xiong C, Monat C, Clark AS, Grillet C, Marshall GD et al. Slow-light enhanced correlated photon pair generation in a silicon photonic crystal waveguide. Opt Lett 2011; 36: 3413–3415. 21886228 10.1364/OL.36.003413

[bib148] Davanço M, Ong JR, Shehata AB, Tosi A, Agha I et al. Telecommunications-band heralded single photons from a silicon nanophotonic chip. Appl Phys Lett 2012; 100: 261104.

[bib149] Matsuda N, Takesue H, Shimizu K, Tokura Y, Kuramochi E et al. Slow light enhanced correlated photon pair generation in photonic-crystal coupled-resonator optical waveguides. Opt Express 2013; 21: 8596–8604. 23571949 10.1364/OE.21.008596

[bib150] Collins MJ, Xiong C, Rey IH, Vo TD, He J et al. Integrated spatial multiplexing of heralded single-photon sources. Nat Commun 2013; 4: 2582. 24107840 10.1038/ncomms3582 PMC3826656

[bib151] Clark AS, Husko C, Collins MJ, Lehoucq G, Xavier S et al. Heralded single-photon source in a III–V photonic crystal. Opt Lett 2013; 38: 649–651. 23455253 10.1364/OL.38.000649

[bib152] Akahane Y, Asano T, Song BS, Noda S. High-Q photonic nanocavity in a two-dimensional photonic crystal. Nature 2003; 425: 944–947. 14586465 10.1038/nature02063

[bib153] Notomi M, Kuramochi E, Taniyama H. Ultrahigh-Q Nanocavity with 1D Photonic Gap. Opt Express 2008; 16: 11095–11102. 18648423 10.1364/oe.16.011095

[bib154] Song BS, Noda S, Asano T, Akahane Y. Ultra-high-Q photonic double-heterostructure nanocavity. Nat Mater 2005; 4: 207–210.

[bib155] Azzini S, Grassani D, Galli M, Gerace D, Patrini M et al. Stimulated and spontaneous four-wave mixing in silicon-on-insulator coupled photonic wire nano-cavities. Appl Phys Lett 2013; 103: 031117.

[bib156] Lai Y, Pirotta S, Urbinati G, Gerace D, Minkov M et al. Genetically designed L3 photonic crystal nanocavities with measured quality factor exceeding one million. Appl Phys Lett 2014; 104: 241101.

[bib157] Ferretti S, Gerace D. Single-photon nonlinear optics with Kerr-type nanostructured materials. Phys Rev B 2012; 85: 033303.

[bib158] Flayac H, Gerace D, Savona V. An all-silicon single-photon source by unconventional photon blockade. Sci Rep 2015; 5: 11223. 26061665 10.1038/srep11223 PMC4462143

[bib159] Volz T, Reinhard A, Winger M, Badolato A, Hennessy KJ et al. Ultrafast all-optical switching by single photons. Nat Photonics 2012; 6: 607–611.

[bib160] Helt LG, Yang ZS, Liscidini M, Sipe JE. Spontaneous four-wave mixing in microring resonators. Opt Lett 2010; 35: 3006–3008. 20847760 10.1364/OL.35.003006

[bib161] Chen J, Levine ZH, Fan JY, Migdall AL. Frequency-bin entangled comb of photon pairs from a Silicon-on-Insulator micro-resonator. Opt Express 2011; 19: 1470–1483. 21263689 10.1364/OE.19.001470

[bib162] Azzini S, Grassani D, Galli M, Andreani LC, Sorel M et al. From classical four-wave mixing to parametric fluorescence in silicon microring resonators. Opt Lett 2012; 37: 3807–3809. 23041866 10.1364/ol.37.003807

[bib163] Mazeas F, Traetta M, Bentivegna M, Kaiser F, Aktas D et al. High-quality photonic entanglement for wavelength-multiplexed quantum communication based on a silicon chip. Opt Express 2016; 24: 28731–28739. 27958516 10.1364/OE.24.028731

[bib164] Sangouard N, Simon C, de Riedmatten H, Gisin N. Quantum repeaters based on atomic ensembles and linear optics. Rev Mod Phys 2011; 83: 33–80.

[bib165] Ong JR, Cooper ML, Gupta G, Green WMJ, Assefa S et al. Low-power continuous-wave four-wave mixing in silicon coupled-resonator optical waveguides. Opt Lett 2011; 36: 2964–2966. 21808373 10.1364/OL.36.002964

[bib166] Kumar R, Ong JR, Recchio J, Srinivasan K, Mookherjea S. Spectrally multiplexed and tunable-wavelength photon pairs at 1.55 μm from a silicon coupled-resonator optical waveguide. Opt Lett 2013; 38: 2969–2971. 24104623 10.1364/OL.38.002969

[bib167] Kumar R, Savanier M, Ong JR, Mookherjea S. Entanglement measurement of a coupled silicon microring photon pair source. Opt Express 2015; 23: 19318–19327. 26367592 10.1364/OE.23.019318

[bib168] Gentry CM, Triginer G, Zeng XG, Popović M. Tailoring of Individual Photon Lifetimes as a Degree of Freedom in Resonant Quantum Photonic Sources. Proceedings of the Conference on Lasers and Electro-Optics: Applications and Technology 2016; 5–10 June 2016; San Jose, CA, USA. Optical Society of America: San Jose, CA, USA 2016.

[bib169] Ong JR, Kumar R, Mookherjea S. Ultra-High-Contrast and Tunable-Bandwidth Filter Using Cascaded High-Order Silicon Microring Filters. IEEE Photonics Technol Lett 2013; 25: 1543–1546.

[bib170] Harris NC, Grassani D, Simbula A, Pant M, Galli M et al. Integrated Source of Spectrally Filtered Correlated Photons for Large-Scale Quantum Photonic Systems. Phys Rev X 2014; 4: 041047.

[bib171] Gentry CM, Shainline JM, Wade MT, Stevens MJ, Dyer SD et al. Quantum-correlated photon pairs generated in a commercial 45 nm complementary metal-oxide semiconductor microelectronic chip. Optica 2015; 2: 1065–1071.

[bib172] He JK, Bell BA, Casas-Bedoya A, Zhang YB, Clark AS et al. Ultracompact quantum splitter of degenerate photon pairs. Optica 2015; 2: 779–782.

[bib173] Helt LG, Steel MJ, Sipe JE. Parasitic nonlinearities in photon pair generation via integrated spontaneous four-wave mixing: Critical problem or distraction? Appl Phys Lett 2013; 102: 201106.

[bib174] Husko CA, Clark AS, Collins MJ, De Rossi A, Combrié S et al. Multi-photon absorption limits to heralded single photon sources. Sci Rep 2013; 3: 3087. 24186400 10.1038/srep03087 PMC3816289

[bib175] Ferrera M, Duchesne D, Razzari L, Peccianti M, Morandotti R et al. Low power four wave mixing in an integrated, micro-ring resonator with Q = 1.2 million. Opt Express 2009; 17: 14098–14103. 19654818 10.1364/oe.17.014098

[bib176] Razzari L, Duchesne D, Ferrera M, Morandotti R, Chu S et al. CMOS-compatible integrated optical hyper-parametric oscillator. Nat Photonics 2009; 4: 41–45.

[bib177] Pasquazi A, Caspani L, Peccianti M, Clerici M, Ferrera M et al. Self-locked optical parametric oscillation in a CMOS compatible microring resonator: a route to robust optical frequency comb generation on a chip. Opt Express 2013; 21: 13333–13341. 23736585 10.1364/OE.21.013333

[bib178] Xiong C, Zhang X, Mahendra A, He J, Choi DY et al. Compact and reconfigurable silicon nitride time-bin entanglement circuit. Optica 2015; 2: 724–727.

[bib179] Reimer C, Kues M, Caspani L, Wetzel B, Roztocki P et al. Cross-polarized photon-pair generation and bi-chromatically pumped optical parametric oscillation on a chip. Nat Commun 2015; 6: 8236. 26364999 10.1038/ncomms9236 PMC4647848

[bib180] Ramelow S, Farsi A, Clemmen S, Luke K, Lipson M et al Monolithic Source of Tunable Narrowband Photons for Future Quantum Networks. Proceedings of the Conference on Lasers and Electro-Optics: QELS_Fundamental Science 2015; 10–15 May 2015; San Jose, CA, USA. Optical Society of America: San Jose, CA, USA 2015.

[bib181] Dutt A, Luke K, Manipatruni S, Gaeta AL, Nussenzveig P et al. On-Chip Optical Squeezing. Phys Rev Appl 2015; 3: 044005.

[bib182] Dutt A, Miller S, Luke K, Cardenas J, Gaeta AL et al. Tunable squeezing using coupled ring resonators on a silicon nitride chip. Opt Lett 2016; 41: 223–226. 26766679 10.1364/OL.41.000223

[bib183] Okawachi Y, Yu MJ, Luke K, Carvalho DO, Lipson M et al. Quantum random number generator using a microresonator-based Kerr oscillator. Opt Lett 2016; 41: 4194–4197. 27628355 10.1364/OL.41.004194

[bib184] Ding X, He Y, Duan ZC, Gregersen N, Chen MC et al. On-Demand Single Photons with High Extraction Efficiency and Near-Unity Indistinguishability from a Resonantly Driven Quantum Dot in a Micropillar. Phys Rev Lett 2016; 116: 020401. 26824530 10.1103/PhysRevLett.116.020401

[bib185] Somaschi N, Giesz V, De Santis L, Loredo JC, Almeida MP et al. Near-optimal single-photon sources in the solid state. Nat Photonics 2016; 10: 340–345.

[bib186] Delteil A, Sun Z, Gao WB, Togan E, Faelt S et al. Generation of heralded entanglement between distant hole spins. Nat Phys 2016; 12: 218–223.

[bib187] Sipahigil A, Jahnke KD, Rogers LJ, Teraji T, Isoya J et al. Indistinguishable Photons from Separated Silicon-Vacancy Centers in Diamond. Phys Rev Lett 2014; 113: 113602. 25259977 10.1103/PhysRevLett.113.113602

[bib188] Christ A, Silberhorn C. Limits on the deterministic creation of pure single-photon states using parametric down-conversion. Phys Rev A 2012; 85: 023829.

[bib189] Migdall AL, Branning D, Castelletto S. Tailoring single-photon and multiphoton probabilities of a single-photon on-demand source. Phys Rev A 2002; 66: 053805.

[bib190] Ma XS, Zotter S, Kofler J, Jennewein T, Zeilinger A. Experimental generation of single photons via active multiplexing. Phys Rev A 2011; 83: 043814.

[bib191] Pittman TB, Jacobs BC, Franson JD. Single photons on pseudodemand from stored parametric down-conversion. Phys Rev A 2002; 66: 042303.

[bib192] Mower J, Englund D. Efficient generation of single and entangled photons on a silicon photonic integrated chip. Phys Rev A 2011; 84: 052326.

[bib193] Kaneda F, Christensen BG, Wong JJ, Park HS, McCusker KT et al. Time-multiplexed heralded single-photon source. Optica 2015; 2: 1010–1013.

[bib194] Mendoza GJ, Santagati R, Munns J, Hemsley E, Piekarek M et al. Active temporal and spatial multiplexing of photons. Optica 2016; 3: 127–132.

[bib195] Xiong C, Zhang X, Liu Z, Collins MJ, Mahendra A et al. Active temporal multiplexing of indistinguishable heralded single photons. Nat Commun 2016; 7: 10853. 26996317 10.1038/ncomms10853 PMC4802115

[bib196] Hong CK, Ou ZY, Mandel L. Measurement of subpicosecond time intervals between two photons by interference. Phys Rev Lett 1987; 59: 2044–2046. 10035403 10.1103/PhysRevLett.59.2044

[bib197] Calkins B, Mennea PL, Lita AE, Metcalf BJ, Kolthammer WS et al. High quantum-efficiency photon-number-resolving detector for photonic on-chip information processing. Opt Express 2013; 21: 22657–22670. 24104153 10.1364/OE.21.022657

