# Supplementary material for: Integrated sources of photon quantum states based on nonlinear optics
Source: Light Sci Appl. 2017 Nov 17;6(11):e17100–. doi: 10.1038/lsa.2017.100 (PMC6062040; doi:10.1038/lsa.2017.100)
Supplement: Supplementary Information [file lsa2017100x1.pdf]

# Supplementary Information

# Integrated sources of photon quantum states based on nonlinear optics

Lucia Caspani,[1,2] Chunle Xiong,[3] Benjamin J. Eggleton,[3] Daniele Bajoni,[4] Marco Liscidini,[5] Matteo Galli,[5] Roberto Morandotti,[6,7,8] and David J. Moss[9]

[1]Institute of Photonics, Department of Physics, University of Strathclyde, Glasgow G1 1RD, UK.

[2]Institute of Photonics and Quantum Sciences, Heriot-Watt University, Edinburgh EH14 4AS, UK.

[3]Centre for Ultrahigh bandwidth Devices for Optical Systems (CUDOS), Institute of Photonics and Optical Science (IPOS), School of Physics, University of Sydney, NSW 2006, Australia.

[4]Dipartimento di Ingegneria Industriale e dell'Informazione, Università di Pavia, via Ferrata 1, 27100, Pavia, Italy.

[5]Dipartimento di Fisica, Università di Pavia, via Bassi 6, 27100 Pavia, Italy.

[6]INRS-EMT, 1650 Boulevard Lionel-Boulet, Varennes, Québec J3X 1S2, Canada.

[7]Institute of Fundamental and Frontier Sciences, University of Electronic Science and Technology of China, Chengdu 610054

[8]National Research University of Information Technologies, Mechanics and Optics, St. Petersburg, Russia.

[9]Center for Microphotonics, Swinburne University of Technology, Hawthorn, Victoria, 3122 Australia.

## A: Definition of quantum superposition and quantum entanglement

*Quantum superposition:* If we consider, for example, a quantum state that can be described by a two-dimensional space (such as a photon polarisation or an electron spin), we find that the quantum state can be described not only as being in state A (e.g., horizontal polarisation or spin up) *or* B (e.g., vertical polarisation or spin down) but also in a superposition of the two:

$$|\psi\rangle = \alpha|A\rangle + \beta|B\rangle, \tag{A.1}$$

where $\alpha$ and $\beta$ are two complex parameters related by the normalisation condition $|\alpha|^2 + |\beta|^2 = 1$. Note that this is conceptually different from the scenario where a system is either in state $A$ with probability $|\alpha|^2$ *or* in state $B$ with probability $|\beta|^2$ (a state known as the mixed state and often represented as $\{|A\rangle; |B\rangle\}$).

*Quantum entanglement:* Superposition also applies to the quantum state of two separate systems (such as two photons and two electrons). These two systems are said to be entangled if their state cannot be described separately, e.g.:

$$|\psi\rangle_{ent} = \alpha|A\rangle_1|A\rangle_2 + \beta|B\rangle_1|B\rangle_2, \tag{A.2}$$

which represents a superposition in which the two systems are in both state A *and* state B. In contrast, a separable state can always be described as the product of the independent states of systems 1 and 2, e.g.:

$$|\psi\rangle_{sep} = |\psi\rangle_1 \otimes |\psi\rangle_2 = (\alpha|A\rangle_1 + \beta|B\rangle_1) \otimes (\gamma|A\rangle_2 + \delta|B\rangle_2)\ . \tag{A.3}$$

**B: Multimode emission and mixed state in heralded single-photon sources**

In SPDC and SFWM, the strong correlations between signal and idler photons determined by momentum and energy conservation can lead to multimode emission. For example, we can consider the frequency correlations between signal and idler photons, where the state can be expressed as:

$$|\psi\rangle_{SPDC/SFMW} = |\omega_1\rangle_s|\omega_{-1}\rangle_i + |\omega_2\rangle_s|\omega_{-2}\rangle_i + |\omega_3\rangle_s|\omega_{-3}\rangle_i + \cdots, \tag{B.1}$$

where frequency pairs $\omega_n$ and $\omega_{-n}$ sum up to the pump frequency for SPDC (or twice the frequency pump for SFWM): $\omega_n + \omega_{-n} = \omega_{pump}$ (or $2\omega_{pump}$). For simplicity, we consider the case of discrete frequencies, as would be the case for SPDC or SFWM in a cavity; however, similar results would also be obtained when taking into account the continuous character of the frequency distribution. Measuring the heralding photon (say the idler) *without resolving its frequency* projects the signal photon into a mixed state $|\psi\rangle_{signal} = \{|\omega_1\rangle; |\omega_2\rangle; |\omega_3\rangle; \ldots\}$. A possible solution is to filter only a single frequency mode so that the state is, e.g., $|\psi\rangle_{SPDC/SFMW} = |\omega_2\rangle_s|\omega_{-2}\rangle_i$. In this case, measuring the heralding idler photon will project the single photon into the pure state $|\omega_2\rangle_s$.

**C: Relation between non-classical correlations and entanglement**

Entanglement and non-classical correlations, which are commonly interchanged, are in fact quite different. While the presence of non-classical correlations is not enough to demonstrate entanglement, the reverse is true – entanglement guarantees the presence of non-classical correlations. To better clarify the difference, we consider a practical example such as the so-called “twin beams”. In this case, as the name suggests, two beams are said to be twins if they display exactly the same intensity at the single-photon level. For example, a laser field impinging on a lossless perfectly balanced 50/50 beam splitter will not generate twin beams; indeed, the intensity at the output ports of the beam splitter is the same only on average. The two beams will exhibit non-correlated intensity fluctuations determined by the quantum nature of light (shot-noise). The amplitude of these fluctuations scales as the inverse square root of the average intensity. On the other hand, in both the SPDC and SFWM processes, in the ideal scenario, the signal and idler beams generated will exhibit the exact same photon statistics. This is intrinsic to the generation process, as one signal photon can be generated if and only if an idler photon is also generated.

Indeed, one means for proving the entanglement of a bipartite system is based on the Peres-Horodecki criterion[1,2]. It defines a necessary condition for separability – its violation is a sufficient (but not necessary) condition for entanglement[3]. However, the violation of this criterion is a sufficient and necessary condition only for 2×2 and 2×3 systems (bipartite two-mode and bipartite tri-mode systems, respectively). We note that the Peres-Horodecki criterion is more sensitive than Bell’s inequalities in the sense that there exist states that are entangled according to the Peres-Horodecki criterion that do not violate any of Bell’s inequalities[3,4].

A version of the Peres-Horodecki criterion for continuous variables was proposed in 2000 by Duan[5] and Simon[6] and shows that for an entangled state the inferred variances of two non-commuting variables, denoted by the operators $\hat{p}$ and $\hat{q}$ (e.g., energy/time, intensity/phase, and position/momentum), violate an inequality of the form:

$$V(\hat{p}_1 - \hat{p}_2) + V(\hat{q}_1 + \hat{q}_2) \geq 2\sqrt{2}, \tag{C.1}$$

where $V(\hat{x}) = \langle\hat{x}^2\rangle - \langle\hat{x}\rangle^2$. Equation (C.1) has been used, for example, to demonstrate the entanglement in the case of twin beams[7,8].

Therefore, in the case of twin beams, entanglement can be demonstrated by showing non-classical correlations between the beam intensities *and* phases[7,8]. However, for well-known systems, such as SPDC and SFWM, for which we know that the origin of the non-classical correlations is indeed entanglement, the existence of these correlations is often considered as an indication of the presence of entanglement. It is also important to stress that non-classical correlations are also used as the basis for quantum metrology, where, for many applications, entanglement is not required since a reduction in the noise of one of the two variables is necessary to achieve higher sensitivity in, for example, high-sensitivity quantum spectroscopy and imaging[9–12].

**References**


1 Peres A. Separability Criterion for Density Matrices. *Phys Rev Lett* 1996; **77**: 1413–1415.

2 Horodecki R. Informationally coherent quantum systems. *Phys Lett A* 1994; **187**: 145–150.

3 Benenti G, Casati G, Strini G. *Principles of quantum computation and information Volume II, Basic Tools And Special Topics*. Singapore: World Scientific; 2007.

4 Popescu S. Bell’s inequalities versus teleportation: What is nonlocality? *Phys Rev Lett* 1994; **72**: 797–799.

5 Duan LM, Giedke G, Cirac JI, Zoller P. Inseparability Criterion for Continuous Variable Systems. *Phys Rev Lett* 2000; **84**: 2722–2725.

6 Simon R. Peres-Horodecki Separability Criterion for Continuous Variable Systems. *Phys Rev Lett* 2000; **84**: 2726–2729.

7 Bowen WP, Schnabel R, Lam PK, Ralph TC. Experimental Investigation of Criteria for Continuous Variable Entanglement. *Phys Rev Lett* 2003; **90**: 043601.

8 Villar AS, Cruz LS, Cassemiro KN, Martinelli M, Nussenzveig P. Generation of Bright Two-Color Continuous Variable Entanglement. *Phys Rev Lett* 2005; **95**: 243603.

9 Nabors CD, Shelby RM. Two-color squeezing and sub-shot-noise signal recovery in doubly resonant optical parametric oscillators. *Phys Rev A* 1990; **42**: 556–559.

10 Tapster PR, Seward SF, Rarity JG. Sub-shot-noise measurement of modulated absorption using parametric down-conversion. *Phys Rev A* 1991; **44**: 3266–3269.

11 Brambilla E, Caspani L, Jedrkiewicz O, Lugiato LA, Gatti A. High-sensitivity imaging with multi-mode twin beams. *Phys Rev A* 2008; **77**: 053807.

12 Brida G, Genovese M, Ruo Berchera I. Experimental realization of sub-shot-noise quantum imaging. *Nat Photonics* 2010; **4**: 227–230.
